# Life cycle complexity and body mass drive erratic changes in climate vulnerability across ontogeny in a seasonally migrating butterfly

**DOI:** 10.1093/conphys/coad058

**Published:** 2023-08-12

**Authors:** Osmary A Medina-Báez, Angie Lenard, Rut A Muzychuk, Carmen R B da Silva, Sarah E Diamond

**Affiliations:** Department of Biology, Case Western Reserve University, 2074 Adelbert Rd, Cleveland, OH 44106, USA; Department of Biology, Case Western Reserve University, 2074 Adelbert Rd, Cleveland, OH 44106, USA; Department of Biology, Case Western Reserve University, 2074 Adelbert Rd, Cleveland, OH 44106, USA; Department of Biology, Case Western Reserve University, 2074 Adelbert Rd, Cleveland, OH 44106, USA; School of Biological Sciences, Monash University, 25 Rainforest Walk, Clayton 3800, Australia; College of Science and Engineering, Flinders University, Anchor Court, Bedford Park 5042, South Australia, Australia; Department of Biology, Case Western Reserve University, 2074 Adelbert Rd, Cleveland, OH 44106, USA

**Keywords:** Developmental trajectory, global change vulnerability, long-distance seasonal migration, thermal physiology, thermal sensitivity, thermal tolerance

## Abstract

Physiological traits are often used for vulnerability assessments of organismal responses to climate change. Trait values can change dramatically over the life cycle of organisms but are typically assessed at a single developmental stage. Reconciling ontogenetic changes in physiological traits with vulnerability assessments often reveals early life-stage vulnerabilities. The degree to which ontogenetic changes in physiological traits are due to changes in body mass over development versus stage-specific responses determines the degree to which mass can be used as a proxy for vulnerability. Here, we use the painted lady butterfly, *Vanessa cardui*, to test ontogenetic changes in two physiological traits, the acute thermal sensitivity of routine metabolic rate (RMR *Q*_10_) and the critical thermal maximum (CT_max_). RMR *Q*_10_ generally followed ontogenetic changes in body mass, with stages characterized by smaller body mass exhibiting lower acute thermal sensitivity. However, CT_max_ was largely decoupled from ontogenetic changes in body mass. In contrast with trends from other studies showing increasing vulnerability among progressively earlier developmental stages, our study revealed highly erratic patterns of vulnerability across ontogeny. Specifically, we found the lowest joint-trait vulnerability (both RMR *Q*_10_ and CT_max_) in the earliest developmental stage we tested (3rd instar larvae), the highest vulnerabilities in the next two developmental stages (4th and 5th instar larvae), and reduced vulnerability into the pupal and adult stages. Our study supports growing evidence of mechanistic decoupling of physiology across developmental stages and suggests that body mass is not a universal proxy for all physiological trait indicators of climate vulnerability.

## Introduction

The responses of physiological tolerance and performance traits to variation in temperature are widely used as indicators of organismal vulnerabilities to global climate change (Huey *et al.,* 2012; Kingsolver *et al.,* 2013; Buckley and Huey, 2016). Organisms with the lowest vulnerability to temperature rise are those with a greater ability to survive exposure to extreme high temperatures or greater plasticity in sub-lethal performance traits such as metabolic rate that allow them to maintain homeostasis in the face of climatic warming (Sunday *et al.,* 2014; Seebacher *et al.,* 2015). Vulnerability assessments are often based on physiological trait values from single developmental stages and are especially biased towards mature stages (Klockmann and Fischer, 2017). Yet, owing to the fact that intra-specific variation in physiological trait values across ontogeny rivals the magnitude of trait differences between species, and can itself be quite variable across taxa (Dahlke *et al.,* 2020, but see Pottier *et al.,* 2022a), the use of single developmental stages for vulnerability assessments can lead to inaccurate estimates of resilience to climate change (Levy *et al.,* 2015). It is increasingly recognized that organisms are only as resilient to climate change as their most vulnerable developmental stage (Klockmann and Fischer, 2017); however, there remain comparatively few tests of ontogenetic changes in physiological traits related to climate vulnerability.

Among ectothermic species, the dominant pattern of stage-dependent vulnerability based on heat tolerance that is emerging in the literature is one of high early-stage vulnerability, followed by reductions in vulnerability towards adolescence and/or metamorphic stages, and sometimes then followed by a resurgence of elevated vulnerability into adulthood. A synthesis of ontogenetic changes in heat tolerance across nearly 700 species of marine and freshwater fish (Dahlke *et al.,* 2020) broadly supports this pattern. No such synthesis currently exists for terrestrial systems, though individual studies of ontogenetic changes in heat tolerance, for example, in the common frog (Ruthsatz *et al.,* 2022), the mealworm beetle (Vorhees and Bradley, 2012), the sirex woodwasp (Li *et al.,* 2019), and a tropical butterfly (Klockmann *et al.,* 2017) provide support for early-stage vulnerability. Using a somewhat different approach, Levy *et al.* (2015) developed a life cycle model of population dynamics for North American lizards based on stage-specific estimates of thermal tolerance and microclimatic variation and found evidence of elevated vulnerability to climate change when including the embryo stage. In terrestrial systems, the pattern of early-stage vulnerability has, in some instances, been attributed to a positive association between body mass and heat tolerance (Chown and Nicolson, 2004; Klockmann *et al.,* 2017).

In comparison to thermal tolerance, very different patterns are evident for thermal sensitivity of metabolic rate, another important physiological trait indicator of climate vulnerability that can change throughout ontogeny (Kingsolver and Buckley, 2020). Organisms with lower thermal sensitivity of metabolic rate are argued to be better able to compensate for changes in environmental temperature and therefore less vulnerable to climatic warming (Seebacher *et al.,* 2015). However, ontogenetic changes in mean metabolic rate can be adaptive. For example, shifts from lower rates in early stages to higher rates in later stages confer benefits to total fitness in a marine bryozoan (Pettersen *et al.,* 2016). Thus, ontogenetic changes in thermal sensitivity of metabolic rate, rather than mean trait values, might provide a more direct link with climate vulnerability (Magozzi and Calosi, 2015). Work in porcelain crabs (Leiva *et al.,* 2018) and the budworm moth (Bawa *et al.,* 2021) shows evidence of lower acute thermal sensitivity, and therefore lower vulnerability in earlier developmental stages compared with later stages. Relatedly, work in dung beetles (Carter and Sheldon, 2020) shows evidence of lower acclimation thermal sensitivity to different chronic temperatures in earlier developmental stages. However, Silva-Garay and Lowe (2021) describe the opposite pattern in stingrays, with juveniles exhibiting higher acclimation thermal sensitivity than adults. Yet caution must be exercised here, as acute and acclimation thermal sensitivities represent different processes (passive versus active plasticity, respectively) and thus cannot be directly compared (Havird *et al.,* 2020). Furthermore, within-stage explorations of the relationship between mass and acute thermal sensitivity of metabolic rate in crickets revealed higher thermal sensitivity with increasing body mass (Nespolo *et al.,* 2003) in certain environmental contexts, lending some support to the between-stage patterns observed among other taxa.

These contrasting patterns of the relationship between ontogeny and climate vulnerability based on heat tolerance versus thermal sensitivity of metabolic rate provide an opportunity to explore potential mechanisms underlying vulnerability across developmental stages. Specifically, they can be used to explore potential roles for: 1) intrinsic stage-dependent changes in physiology, shaped by stage-specific exposure to seasonal and microclimatic variation in temperature (Kingsolver *et al.,* 2011), and reinforced by evidence of decoupling of physiological tolerance and performance mechanisms across ontogeny (Freda *et al.,* 2017, 2022), 2) ontogenetic changes in body mass and downstream effects on physiology (Klockmann *et al.,* 2017), and 3) stage-specific changes in activity driven by inherent mobility differences (e.g. relatively immobile pupae versus mobile adults), particularly for effects on metabolism (Carter and Sheldon, 2020). Here we used the painted lady butterfly, *Vanessa cardui*, to examine how high temperature tolerance and acute thermal sensitivity of metabolic rate change across ontogeny, from the 3rd larval instar through the 5th larval instar, and at the pupal and adult stages. If greater body mass is important for driving reduced vulnerability across ontogeny, we expected heat tolerance to increase with developmental stage from 3rd instar up to pupation and then decrease at the adult stage. By contrast, if lower body mass is important for driving reduced vulnerability across ontogeny, we expected acute thermal sensitivity of metabolic rate to increase with developmental stage (excepting the immobile pupal stage). Alternatively, if body mass is not responsible for driving vulnerability, we expected to find idiosyncratic changes in vulnerability across developmental stages. We further explored within-stage patterns of the relationship between mass and physiological traits, with the expectation that these would follow the between-stage patterns. Finally, we examined the effects of developmental acclimation temperature (20 versus 30°C) on between-stage and within-stage patterns to explore whether ontogenetic patterns were dependent on environmental context, as higher developmental acclimation temperatures can enhance the ability to physiologically resist acute thermal challenges (Angilletta, 2009; Havird *et al.,* 2020).

## Methods

### Study system

The painted lady butterfly, *V. cardui* (Lepidoptera: Nymphalidae) is a holometabolous insect that goes through complete metamorphosis, with discrete developmental stages including five larval instars, and pupal and adult stages. This species has a nearly global distribution (excluding Antarctica and South America) and undergoes seasonal migration in temperate regions (Abbott, 1951; Stefanescu *et al.,* 2013). *Vanessa cardui* uses long-distance movement to stay within suitable climatic and resource niches (Hu *et al.,* 2021), making this species sensitive to changes in environmental temperature (Poston *et al.,* 1977; Kelly and Debinski, 1999), despite its widespread geographic distribution.

### Experimental design and laboratory rearing

We performed a laboratory experiment to quantify: 1) how two physiological indicator traits of vulnerability to warming change throughout ontogeny, and 2) the effects of developmental acclimation temperature on these ontogenetic changes in vulnerability. Our two physiological traits were the critical thermal maximum and the acute thermal sensitivity of metabolic rate.

To quantify responses across all developmental stages from the third larval instar to the adult stage, the experiment was conducted in two phases. Phase 1 included measurements on 3rd and 5th larval instars, pupae (acute thermal sensitivity of metabolic rate only), and adults (critical thermal maximum only) and ran from 11 March to 26 May 2021. Phase 2 included measurements on 3rd and 4th larval instars, and adults, and ran from 3 September to 22 November 2021. Measurements for 3rd instar larvae and adults were used to ensure congruence of results across phases 1 and 2 of the experiment.

We set up 32 individual larvae (3rd instar) of *V. cardui* (Carolina Biological) to establish the adult mating pairs whose offspring were measured in each of the two phases of the physiological trait experiment. Animals used to establish the mating pairs were held at a constant 25°C with a 14:10 L:D photoperiod (Percival Scientific growth chamber, growth chamber, 36-VL). Mating pairs were kept in flight cages (30cm each dimension: l × w × h, BugDorm) on benchtops in the laboratory near natural light. Mating pairs were provided with continuous access to food and water (10% sucrose solution) and a water-moistened paper towel as a substrate for oviposition.

The eggs produced from each mating pair (hereafter, ‘family’) were then split across two developmental acclimation temperature treatments (constant 20°C or constant 30°C, each on a 14:10 L:D photoperiod). Eggs were randomly assigned to the temperature treatments. Larvae were housed individually in small plastic cups (118 mL) and were provided with continuous access to an artificial diet (Carolina Biological painted lady butterfly culture medium). Larval molts were determined based on the presence of a shed head capsule and cuticle. Larvae were allowed to metamorphose in their larval rearing cups. Following pupation, animals were housed individually in larger plastic cups (500 mL) until adult eclosion.

At each developmental stage, beginning with the 3rd larval instar through the adult stage, we removed a subset of animals for physiological trait assessment. Animals were assigned a random number for physiological trait assessment in an effort to blind the researcher to the temperature treatment from which animals were taken. However, the effects of temperature on development time and an inability to mask the developmental stage of the animal during trait assessment prevented complete blinding of subject identity in our experiment. We first assessed metabolic rate at two test temperatures to quantify the acute thermal sensitivity of metabolic rate. Following metabolic rate measurements, we then assessed the critical thermal maximum on these same animals. Because the critical thermal maximum assay is often lethal, we do not have repeated assessments of the physiological traits across ontogeny for a single individual, but rather a sample of individuals (from the same family) selected for assessment at a particular developmental stage. We aimed to assess physiological traits (both the critical thermal maximum and acute thermal sensitivity of metabolic rate) for a minimum of 30 animals at each stage, comprising a median of 3 individuals per family (see Supplementary Material, Table S1 for a summary of sample sizes for physiological traits at each stage).

### Metabolic rate

To assess the thermal sensitivity of metabolic rate, we measured the metabolic rate of individuals at two acute test temperatures, 20 and 30°C. Because we measured metabolic rate while the animal was permitted to engage in normal behaviours, we define our metabolic rate measure as ‘routine’ metabolic rate (RMR; *sensu*  Metcalfe *et al.,* 2016). To quantify metabolic rate at each test temperature, we used a CO_2_/H_2_O gas analyser (LI-7000, LI-COR Biosciences) in push mode that pushed air from the environment (scrubbed of CO_2_ and H_2_O with soda lime and Drierite, Sigma Aldrich) through two flow control meters (Alicat Scientific, MC-200SCCM for phase 1 of the experiment and MC-1SLPM for phase 2; this difference in flow control meters is due to the fact that adults, which require larger respirometry chambers, were only assessed for metabolic rate during phase 2 of the experiment) and then a respirometry flow multiplexer (RM-8, Sable Systems International). The respirometry equipment was held within a dark growth chamber (MIR 154, PHCbi) set to a constant 20 or 30°C. The flow controllers were calibrated (Gilian Gilibrator-2 Calibrator, Sensidyne LP) at both 20 and 30°C. Animals were tested at 20 and 30°C in a random order. Once placed inside the respirometry chambers, animals were allowed to acclimate at the given test temperature for 15 minutes prior to recording of metabolic rate.

We tested larvae (3rd to 5th instars) and pupae inside 30 mL glass chambers, and adults in 650 mL glass chambers (RC and RC-1; Sable Systems International). Eggs and very early stage larvae (1st and 2nd instars) were too small to reliably obtain respirometry recordings in preliminary trials, and so were omitted from the design. In the multiplexer, each animal chamber (n = 8 chambers maximum) contained one individual. CO_2_ was recorded for 10 minutes, with a 2-minute flush, at a constant flow rate, adjusted separately for each test temperature to achieve a volumetric flow rate of 100 mL min^−1^. For adults, the larger respirometry chambers required a higher flow rate, adjusted to achieve a target volumetric flow rate of 500 mL min^−1^, and a longer flush of 14 minutes. CO_2_ concentration (ppm) from the animal chambers was compared with the CO_2_ concentration (ppm) from the returning control line and recorded by the Licor-7000. All of the metabolic rate data were processed through a UI-3 data acquisition interface and ExpeData software (Sable Systems International). Once flow rate calibrations were complete, we then converted the raw CO_2_ values to the rate of CO_2_ production (VCO_2_ mL min^−1^) by dividing the outputted values by 1 000 000 to get the fractional CO_2_ value and multiplied this value by the flow rate. We considered the first 5 minutes as a settling-in period, and used the last 5 minutes of recording to compute the mean metabolic rate over this period. These butterflies breathe continuously and so the 5 minute interval is sufficient to get an estimation of metabolic rate, unlike species with discontinuous breathing (Winwood-Smith and White, 2018). We performed a complementary method to detect the flattest part of the trace using a rolling window analysis (each 5-minute interval possible over the 10 minutes of recording) and identifying the lowest slope value. Because calculations of metabolic rate based on this rolling window method were nearly identical to the last 5 minutes recording approach, we elected to use the latter method for simplicity. Finally, we standardized the change in metabolic rate across the two test temperatures as *Q*_10_ values, which describe the increase in metabolic rate for every 10°C increase in temperature (Birk, 2021), yielding our focal metric of acute thermal sensitivity of routine metabolic rate (RMR *Q*_10_).

For larvae, metabolic rate was assessed within 24 hours of the molt to the new instar. Larvae typically molted overnight, and metabolic rate was assessed the next day. During this period, the molting fluid was allowed to evaporate and the cuticle allowed to harden. On the morning of metabolic rate trials, post-molt larvae were sorted into cups without food prior to the assessment of metabolic rate. For adults, metabolic rate was assessed 24–48 hours after eclosion to allow the wings to expand and dry. Adults were kept at their respective developmental acclimation temperature during this period. Further, adults were not provided access to sugar solution prior to the assessment of metabolic rate, as the time since last feeding is especially influential for the metabolic rate of adult *V. cardui* (Woods Jr *et al.,* 2010).

### Critical thermal maximum

Following assessment of metabolic rate at both the 20 and 30°C test temperatures, individuals were allowed to recover for a 15-minute period on the laboratory bench (~23°C, 30–40% relative humidity, RH) prior to assessment of the critical thermal maximum (CT_max_). The last test temperature experienced during the metabolic rate trial did not have a significant effect on CT_max_ (3rd instar: *F*_1,78_ = 0.510, *P* = 0.477; 4th instar: *F*_1,54_ = 1.26, *P* = 0.266; 5th instar: *F*_1,46_ = 0.378, *P* = 0.542; Adult: *F*_1,49_ = 0.763, *P* = 0.387, after accounting for the effects of developmental acclimation temperature and body mass), so we pooled data across those animals that experienced acute temperatures of 20°C versus 30°C most recently prior to the assessment of CT_max_. The CT_max_ trials were performed using a water bath (A40 ARCTIC SC150, Thermo Fisher Scientific) with a dynamic temperature ramping protocol of 1°C min^−1^. The CT_max_ was designated as the temperature at which complete loss of movement occurred (i.e. when turning the containers over yielded no movement response from the organism), as this was a consistent diagnostic feature across all mobile developmental stages. We elected to use dynamically ramped assays for CT_max_ in an effort to limit the confounding effects of starvation, hydration, and thermal acclimation, particularly since these confounding effects might be sensitive to ontogenetic changes in mass (Terblanche *et al.,* 2011). However, this assay approach relies on behaviour (loss of movement) and could not be applied to immobile stages of eggs and pupae, nor very early instar larvae (1st and 2nd instar) that were too small to reliably observe loss of movement. For the assessment of CT_max_, larvae were housed in 12 mL plastic test tubes with a cotton plug. Adults were tested using 200 mL plastic containers plugged with a sponge. At the start of the trial, all individuals were placed in individual containers, and were allowed to acclimate to the starting water bath temperature of 35°C (starting RH being the same as room RH, ~ 30–40%) for 15 minutes.

### Developmental trajectories, pre-trial body mass, and differences between sexes

We quantified developmental trajectories from the third larval instar through the adult stage by recording body mass and age at each developmental stage upon the molt to the new instar, pupation, or adult eclosion. Body mass was recorded to a precision of 0.0001 g (MSE124S-100-DA; Sartorius). These measurements also provided estimates of body mass prior to the assessment of physiological traits for the subset of animals removed for testing at each developmental stage. Note that because we recorded age and mass at each developmental stage prior to the given stage selected for physiological trait testing, we have comparatively greater sample sizes for developmental trajectory components (Supplementary Material, Tables S2, S3) than for the physiological trait measurements.

Sexual size dimorphism is generally marginal in *V. cardui* (O’Neill *et al.,* 2008). We were able to determine the sex of most individuals at the pupal stage based on an abdominal suture that is present in females and absent in males (Genc, 2005). This diagnostic character was ambiguous for a small fraction of individuals that could not be sexed (12 out of 126 pupae that underwent physiological trials at the pupal stage and 22 out of 129 pupae that underwent trials at the adult stage). For those animals where sex could be determined, we were able to test for sexual size dimorphism in the pupal and adult stages. This also allowed us to directly model the effects of sex on physiological traits. However, for the larval instars, data were necessarily pooled across sex when examining the relationship between physiological traits and body mass.

### Statistical analyses

We performed all statistical analyses using R version 4.2.1 (R Core Team, 2022). We present all results to 3 significant digits. To address our focal question of how climate vulnerability traits vary across developmental stage, we constructed a series of related models.

First, we examined developmental trajectories across ontogeny to establish a baseline expectation for how vulnerabilities should change with stage under an assumption of body mass as a main driver of stage-dependent vulnerability. To accomplish this, we constructed a linear mixed effects model using the *lme* function from the *nlme* library (Pinheiro *et al.,* 2022) to quantify how body mass varies across ontogeny under different developmental acclimation temperatures. We considered body mass (natural-log transformed) as the response, and developmental stage, developmental acclimation temperature, and their interaction as the predictor variables. Family identity was included as a random intercept.

For our focal analyses, we then constructed several linear mixed effects models to examine how physiological traits vary across ontogeny. Either CT_max_ or RMR *Q*_10_ was considered as the response variable. We included developmental stage, developmental acclimation temperature, and their interaction as predictors. We performed two subsets of models that either additionally included or excluded body mass, taken prior to physiological trait testing and natural log transformed, as a covariate. The models that excluded body mass as a covariate (hereafter “mass-dependent” models of CT_max_ or RMR *Q*_10_) allowed us to assess the ecologically relevant patterns of vulnerability across developmental stages. The models that included body mass as a covariate (hereafter “mass-independent” models of CT_max_ or RMR *Q*_10_) allowed us to assess the degree to which stage-dependent changes in body mass were responsible for ontogenetic changes in physiological trait values and climate vulnerability (following Downs *et al.,* 2013 for comparisons of mass-dependent and mass-independent models of physiological traits, and Nespolo *et al.,* 2003 for inclusion of mass as a covariate in models of metabolic rate *Q*_10_). Statistical significance of the developmental stage term (and the associated pairwise contrasts between developmental stages) with body mass included as a covariate in the model would indicate an important role for factors other than mass in driving differences in vulnerability across developmental stages. We treated body mass as a simple covariate in these models, rather than interacting body mass with acclimation temperature and developmental stage, as here we were interested in the between-stage patterns. Family identity was included as a random intercept.

We used type III (marginal) *F*-tests to examine the statistical significance of model predictors. In the case where significant effects of developmental stage were found, we used pairwise post-hoc comparisons from the *emmeans* library (Lenth, 2022) to determine which stages were different from one another.

We also examined within-stage relationships between body mass and physiological traits. In this case, we performed models as above for comparisons across stages, but allowed the slope of the body mass-physiological trait relationship to vary by stage and developmental acclimation temperature (i.e. we included a 3-way interaction between mass, temperature, and stage). We then used the *emtrends* function from the *emmeans* library to examine the relationship between body mass and physiological trait values (either CT_max_ or RMR *Q*_10_) at a given developmental stage and for each of the two developmental acclimation temperatures.

For within-stage analyses, we were able to explore the effects of sex on physiological traits at the pupal and adult stages. We constructed models on subsets of the data for either pupal or adult stages. For adults, we examined both CT_max_ and RMR *Q*_10_ as functions of developmental acclimation temperature, mass, sex, and up to their three-way interaction. For pupae, we examined RMR *Q*_10_ only, but with this same set of predictor variables. Family identity was included in each of these models as a random intercept. When the sex term was not significant, we removed this term from the model to allow for more direct comparisons with larval within-stage model results.

We further used within-stage comparisons to explore the relationship between CT_max_ and RMR *Q*_10_. Owing to our experimental design with repeated measures of the two physiological traits on the same individual, we were able to explore potential patterns of covariation among these traits within a given developmental stage. To do so, we first computed the residuals from linear mixed effects models for each of the two physiological traits that accounted for the effects of body mass and developmental acclimation temperature and for family-level autocorrelation. We then examined the Spearman rank correlation between CT_max_ and RMR *Q*_10_ for each developmental stage.

Finally, because the experiment was conducted in two phases, we assessed whether there were differences between comparable developmental stage responses performed during both experiment phases. For the 3rd larval instar and adult stage, we developed a model of CT_max_ as the response, experiment phase (a two-level factor corresponding to phases 1 and 2 of the experiment) and developmental stage as predictors, and family identity as a random intercept.

## Results

**Figure 1:**
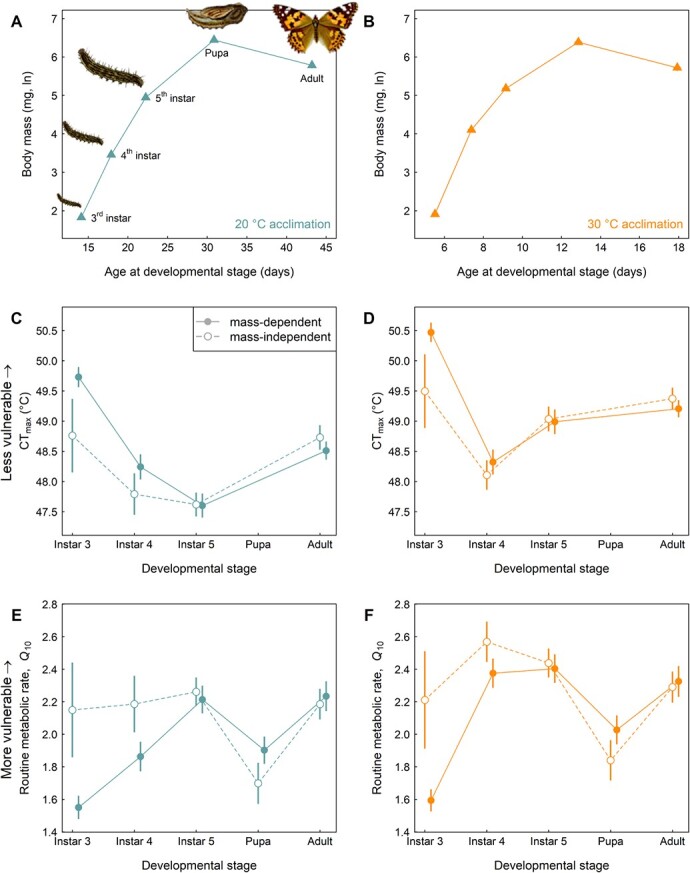
Developmental trajectories set expectations of mass-dependent vulnerability to temperature in the context of CT_max_ and RMR *Q*_10_. Trajectories (mean body mass ± 1 SE, natural log transformed, as a function of mean age at developmental stage ±1 SE; Supplementary Material, Table S3) are provided for the two developmental acclimation temperatures separately, including (A) 20°C and (B) 30°C. Note that SEs are largely not visible owing to their small amount relative to the point size depicting the mean values. Under the assumption of mass-dependent vulnerability, pupae and adults would be expected to exhibit the least vulnerability. Panels (C–F) show the actual stage-dependent results for physiological trait vulnerability estimates. Data are presented both as mass-dependent estimates (filled symbols, solid lines) and mass-independent estimates (open symbols, dashed lines). Predicted critical thermal maximum (CT_max_) values ±1 SE from a linear mixed effects model of CT_max_ as a function of the interaction of developmental stage and developmental acclimation temperature, a random intercept for family identity, and, for the mass-independent estimates, a covariate of body mass, are plotted as a function of developmental stage. Note that CT_max_ values could not be obtained for the pupal stage. CT_max_ data are plotted separately for (C) 20°C and (D) 30°C developmental acclimation temperatures. Predicted routine metabolic rate (RMR) *Q*_10_ values ±1 SE from a linear mixed effects model of RMR *Q*_10_ as a function of the interaction of developmental stage and developmental acclimation temperature, a random intercept for family identity, and, for the mass-independent estimates, a covariate of body mass, are plotted as a function of developmental stage. RMR *Q*_10_ data are plotted separately for (E) 20°C and (F) 30°C developmental acclimation temperatures. Drawings of the developmental stages in panel A were obtained from William Buckler’s The larvæ of the British butterflies and moths, and Jacob Hübner’s Das kleine Schmetterlingsbuch (both public domain).

Our analyses of ontogenetic changes in body mass and age at each developmental stage of *V. cardui* revealed significant differences between each stage (Fig. 1A,B; Supplementary Material, Table S2; all pairwise contrasts between developmental stage for body mass and age at a given stage were significant, *P* < 0.0001). For both the 20 and 30°C developmental acclimation temperature treatments, the rank order of body mass (measured at the beginning of each stage) from smallest to largest was 3rd instar, 4th instar, 5th instar, adult and pupa (Fig. 1A,B; Supplementary Material, Table S3).

### Between-stage patterns of vulnerability

Because we did not detect significant differences in physiological trait values (CT_max_ at 3rd instar and adult stages) between phases 1 and 2 of the experiment (*F*_1,14_ = 0.500, *P* = 0.493), we combined the two datasets for analysis. We found significant differences in vulnerability across ontogeny, though importantly, not all of these differences were attributable to ontogenetic changes in mass. Rather, some were due to stage-specific changes in physiological trait values independent of mass. That is, even with mass as a covariate in the models, significant differences in vulnerabilities between some developmental stages were detected (Table 1; Fig. 1C–F).

**Table 1:** Statistical significance (test statistics and *P*-values) of predictors from linear mixed effects models of physiological traits, CT_max_ and RMR *Q*_10_, as a function of developmental stage, developmental acclimation temperature and their interaction

Response	Model form	Term	*F*	*P*
CT_max_	Mass-dependent	Acclimation	12.8	**0.000399**
		Stage	6.18	**0.000423**
		Acclimation × stage	4.19	**0.00623**
	Mass-independent	Body mass	2.82	0.094
		Acclimation	12.7	**0.000419**
		Stage	4.06	**0.00743**
		Acclimation × stage	3.14	**0.0256**
RMR *Q*_10_	Mass-dependent	Acclimation	0.243	0.622
		Stage	15.9	**<0.0001**
		Acclimation × stage	3.03	**0.0179**
	Mass-independent	Body mass	4.62	**0.0324**
		Acclimation	0.488	0.485
		Stage	6.60	**<0.0001**
		Acclimation × stage	1.09	0.363

Results presented separately for models where body mass was either excluded as a covariate (mass-dependent models) or included as a covariate (mass-independent models). Significant effects (*P* ≤ 0.05) are indicated with bolded *P*-values. For mass and acclimation temperature, ndf = 1; for stage and its interaction with acclimation temperature, ndf = 3 for CT_max_ models and ndf = 4 for RMR *Q*_10_ models. For CT_max_, ddf = 336 for the mass-dependent model and ddf = 335 for the mass-independent model For RMR *Q*_10_, ddf = 325 for the mass-dependent model and ddf = 324 for the mass-independent model.

Overall, mass-dependent CT_max_ was highly erratic across ontogeny, and was influenced by developmental acclimation temperature (Fig. 1C,D; Supplementary Material, Table S4). Warmer developmental acclimation temperature increased CT_max_, but acclimation temperature also interacted with developmental stage. In particular, all pairwise contrasts for CT_max_ under the 20°C developmental acclimation temperature were significant except for between 4th instar larvae and adults, and between 4th and 5th instar larvae. By comparison, under the 30°C acclimation temperature, while non-significant pairwise contrasts were again detected between 4th and 5th instar larvae, the difference between 5th instar larvae and adults was non-significant.

**Figure 2:**
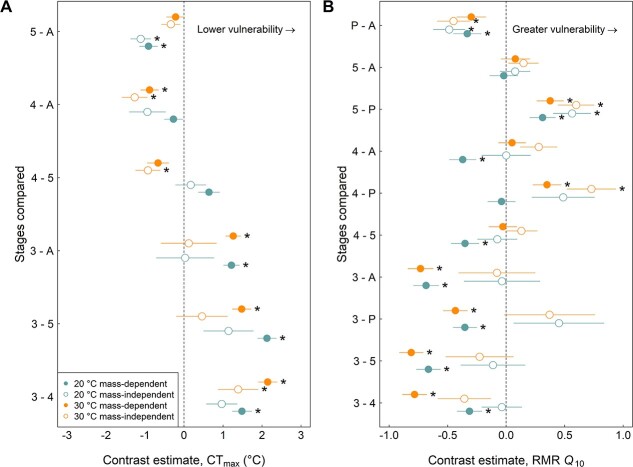
Pairwise post-hoc comparisons for the differences in physiological traits between developmental stages for a given developmental acclimation temperature. Contrast estimates ±1 SE are shown for (A) CT_max_ and (B) RMR *Q*_10_, including results from mass-dependent (filled symbols) and mass-independent (open symbols) models and for 20°C and 30°C developmental acclimation temperature treatments (bottom two versus top two points, respectively). Contrasts are given such that the later developmental stage is subtracted from the earlier stage. Stages are abbreviated on the *y*-axis tick labels: 3, 4 and 5 correspond with their respective larval instars, P indicates pupae and A indicates adults. Asterisks to the right of each contrast indicate statistical significance (*P* ≤ 0.05).

These patterns of interactive effects between acclimation temperature and developmental stage were even more pronounced for the mass-independent models (Fig. 2A; Supplementary Material, Table S5). While 4th instar larvae had significantly lower CT_max_ than 3rd instar larvae when reared under the 30°C acclimation temperature treatment, this effect was non-significant under the 20°C acclimation treatment. Further, while 4th instar larvae were significantly less tolerant than both 5th instar larvae and adults under the 30°C acclimation treatment, these contrasts were not significant under the 20°C acclimation treatment. Instead, under the 20°C acclimation treatment, 5th instar larvae were significantly less tolerant than adults.

While the overall trends for vulnerability across ontogeny were similar in the mass-independent models as the mass-dependent models, in mass-independent models the differences at later larval instars were more subtle, and the difference between 3rd instar larvae and adults was no longer significant. In the mass-independent models, CT_max_ was generally greatest during the 3rd larval instar, lowest during the final two instars (4th and 5th instar), and intermediate during the adult stage (Supplementary Material, Tables S2, S5; Fig. 1C,D). Both the fact that mass-independent models still indicate lower vulnerability based on CT_max_ for 3rd instar larvae, and that the general pattern of stages with smaller body mass (3rd instar) exhibiting higher CT_max_, and stages with larger body mass (5th instar) exhibiting lower CT_max_, indicates decoupling between stage-driven differences in mean body mass and CT_max_.

For mass-dependent RMR *Q*_10_, vulnerability appeared to generally track ontogeny in the larval stages with 3rd instar being least vulnerable (i.e. the lowest acute thermal sensitivity of metabolic rate), followed by an increase in vulnerability in the 4th instar, and even greater vulnerability in the 5th instar (Fig. 1E, F). As with CT_max_, there was evidence of an interaction between stage and developmental acclimation temperature for these larval stage comparisons, as 4th instar larvae were less vulnerable compared with 5th instar larvae under the 20°C acclimation temperature, but not under the 30°C acclimation temperature. At later developmental stages, RMR *Q*_10_ of pupae and adults was significantly elevated compared with 3rd instar larvae at both acclimation temperatures (Fig. 2B; Supplementary Material, Table S4).

As further evidence that mass was a major driver of ontogenetic variation in RMR *Q*_10_, mass-independent models did not indicate significant differences between any of the three larval instar stages (Fig. 2B; Supplementary Material, Table S5). Similarly, the significantly lower acute thermal sensitivity of metabolic rate in 3rd instar versus pupae and adults detected in the mass-dependent models became non-significant in the mass-independent models. For mass-independent models, only pupae (i.e. the only non-mobile stage) had consistently lower RMR *Q*_10_ values compared with adults and with 5th instar larvae across the two developmental acclimation temperatures. Pupae also had lower RMR *Q*_10_ than 4th instar larvae, but only at the 30°C acclimation temperature.

### Within-stage relationships

**Figure 3:**
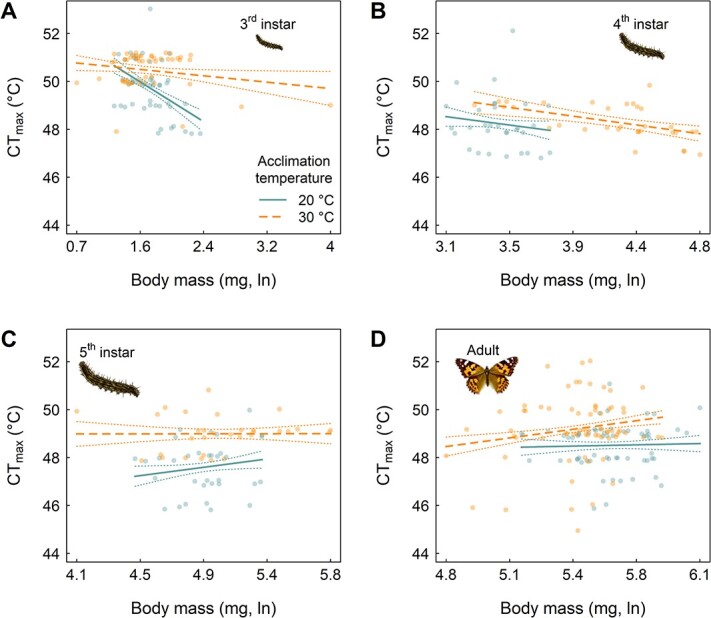
Within-stage patterns of the relationship between CT_max_ and body mass. Panels show results for each developmental stage for which CT_max_ was measured: (A) 3rd instar, (B) 4th instar, (C) 5th instar and (D) adult. Note the common *y*-axis range across panels for CT_max_, but different *x*-axis ranges for body mass. Raw data points (actual *x*-values are shown, whereas the *y*-values being integers required a small amount of random jittering [in the *y*-direction only with a maximal shift of 0.2°C in either the positive or negative direction] to aid in visualization) are overplotted with predicted slopes (solid lines) ± 1 SE (dotted lines) from a linear model of CT_max_ (°C) as a function of developmental stage, developmental acclimation temperature, body mass (mg, natural log transformed), and up to their three-way interaction are presented. Raw, un-jittered data are provided online (see Data Availability statement). Results from the 20°C developmental acclimation temperature treatment are shown in solid lines, and from the 30°C developmental acclimation temperature treatment in dashed lines.

The relationship between body mass and physiological traits appeared to differ strongly among stages when considering CT_max_, but not RMR *Q*_10_. For RMR *Q*_10_, there was only one significant relationship between RMR *Q*_10_ and body mass within each developmental stage and for each developmental acclimation temperature (Table 2). By contrast, for CT_max_, there were several significant relationships between CT_max_ and body mass, with qualitatively different relationships across the developmental stages (Fig. 3). In the 3rd larval instar, large body mass conferred significantly lower CT_max_ among individuals reared under the 20°C acclimation temperature. This negative relationship between mass and CT_max_ was also detected in the 4th instar, but the magnitude of the effect was weaker, and only significant among individuals reared under the 30°C acclimation temperature. By the 5th larval instar, the relationship between mass and CT_max_ was not significant among individuals reared at either of the two acclimation temperatures. At the adult stage, there was a significant positive relationship between mass and CT_max_, but only among individuals reared under the 30°C acclimation temperature. We did not detect any significant effects of sex in the models of within-stage relationships between body mass and either of the two physiological traits (CT_max_ or RMR *Q*_10_) for the pupae or adults where sex could be determined (Supplementary Material, Table S6). We therefore dropped the term of sex from further consideration.

**Table 2:** Estimates (slopes), standard errors, test statistics and *P*-values from post-hoc analyses of linear mixed effects models of within-stage patterns of the relationship between each of the two physiological traits, CT_max_ and RMR *Q*_10_ and body mass (natural log transformed)

Response	Acclimation temperature	Stage	Slope estimate	SE	*t*	*P*
CT_max_	20°C	3rd instar	−2.03	0.592	−3.43	**0.000678**
		4th instar	−0.810	0.973	−0.833	0.406
		5th instar	0.828	0.818	1.01	0.312
		Adult	0.155	0.644	0.240	0.810
	30°C	3rd instar	−0.326	0.296	−1.10	0.271
		4th instar	−0.848	0.433	−1.96	**0.0508**
		5th instar	0.00848	0.513	0.0173	0.987
		Adult	1.05	0.524	2.00	**0.0463**
RMR *Q*_10_	20°C	3rd instar	0.181	0.161	1.13	0.26
		4th instar	−0.00946	0.436	−0.0217	0.983
		5th instar	0.0807	0.351	0.23	0.818
		Pupa	−0.425	0.508	−0.835	0.404
		Adult	0.119	0.465	0.256	0.798
	30°C	3rd instar	0.185	0.169	1.09	0.277
		4th instar	0.495	0.191	2.6	**0.00981**
		5th instar	0.0927	0.21	0.442	0.659
		Pupa	−0.346	0.391	−0.883	0.378
		Adult	0.0695	0.51	0.136	0.892

Slopes that are significantly different from zero (*P* ≤ 0.05) have bolded *P*-values. For the CT_max_ post-hoc analyses, the ddf = 328; for RMR *Q*_10_ post-hoc analyses, the ddf = 315.

The repeated measures aspect of our study allowed us to examine the individual-level associations of CT_max_ and RMR *Q*_10_. We detected a significant negative relationship between CT_max_ and RMR *Q*_10_ early in development (3rd larval instar). That is, an individual with low vulnerability based on CT_max_ also had low vulnerability based on RMR *Q*_10_ within the 3rd instar (Table 3). However, this pattern weakened over development and became non-significant by the adult stage.

**Table 3:** Correlations (Spearman’s rank correlation coefficient, test statistic, *P*-value, and sample size) between residual CT_max_ and RMR *Q*_10_ after accounting for the effects of body mass and developmental acclimation temperature. Correlations significantly different from zero (*P* ≤ 0.05) have bolded *P*-values

Stage of comparison	n	rho	*S*	*P*
3rd instar	101	−0.34	230 000	**0.000504**
4th instar	63	−0.201	50 000	0.114
5th instar	64	−0.199	52 400	0.114
Adult	58	0.0456	31 000	0.733

## Discussion

Thermal physiological traits often change dramatically across ontogeny, causing different developmental stages to become more vulnerable to climatic warming than others. Yet, depending on the particular physiological traits used to assess vulnerability, contrasting predictions can be made for how body mass and developmental stage might influence physiological trait values. Heat tolerance (CT_max_) might increase with gains in body mass over the life cycle, leading to reduced vulnerability. Acute thermal sensitivity of routine metabolic rate (RMR *Q*_10_) might also increase with gains in body mass over the life cycle, but with the effect of elevating vulnerability. We tested these contrasting predictions using the painted lady butterfly, *V. cardui*. We compared mass-dependent and mass-independent changes in vulnerability across developmental stage to understand how much of the variation in patterns of vulnerability across ontogeny was driven by changes in body mass versus stage-specific responses independent of mass. We found that while RMR *Q*_10_ was largely structured by ontogenetic changes in mass, CT_max_ was decoupled from ontogenetic changes in mass. The combination of expected ontogenetic changes for RMR *Q*_10_ and unexpected changes for CT_max_ contributed to erratic patterns of vulnerability across ontogeny. Our results suggest that body mass is not always a suitable proxy of stage-dependent physiological vulnerability to climate, and that assumptions of progressively increasing vulnerability with earlier developmental stages are not necessarily valid.

### A rugged landscape of ontogenetic changes in vulnerability

Studies in terrestrial ectotherms provide evidence of elevated vulnerability to climate at earlier developmental stages based on heat tolerance (Vorhees and Bradley, 2012; Klockmann *et al.,* 2017; Li *et al.,* 2019; Ruthsatz *et al.,* 2022) and thermal sensitivity of metabolic rate (Leiva *et al.,* 2018; Carter and Sheldon, 2020; Bawa *et al.,* 2021). The general consensus among these studies is that the rank-order vulnerability tends to continue to increase among progressively earlier developmental stages, and is in large part driven by ontogenetic changes in body mass (this pattern is most evident prior to metamorphosis, since post-metamorphic mass loss can generate a small increase in vulnerability; see especially Klockmann *et al.,* 2017; Ruthsatz *et al.,* 2022). By contrast, our results indicate much more erratic changes in vulnerability across ontogeny in the painted lady butterfly. We found the lowest joint-trait vulnerability (considering both CT_max_ and RMR *Q*_10_) in the 3rd larval instar, followed by the highest vulnerability in the 4th and 5th larval instars, and finally followed by a reduction in vulnerability (though not to the level of 3rd instar larvae) in the metamorphic pupal stage and final adult stage (Fig. 1C–F). Importantly, these patterns appeared to be underlain by different mechanisms for different traits and different developmental stages. Ontogenetic variation in RMR *Q*_10_ was driven by a combination of stage-dependent changes in body mass and activity levels. Specifically, larger body mass was generally associated with higher RMR *Q*_10_ values (Fig. 1A,B,E,F). Further, despite the large body mass of pupae, their immobility likely drove their relatively low RMR *Q*_10_ values (e.g. similar to Carter and Sheldon, 2020). By contrast, CT_max_ was largely independent of ontogenetic changes in body mass. Most notably, 3rd instar larvae had the smallest body mass, but highest CT_max_ values (Fig.l 1A–D).

Within-stage patterns for the relationship between body mass and CT_max_ lent further support to the interpretation of highly idiosyncratic changes in CT_max_ across ontogeny. In particular, individuals with large body mass exhibited a cost to CT_max_ at the 3rd larval instar (similar to many aquatic arthropods, e.g. Brans *et al.,* 2017, Leiva *et al.,* 2019, and some terrestrial arthropods, e.g. Youngblood *et al.,* 2019). However, this pattern qualitatively changed by the adult stage, with individuals with large body mass exhibiting higher CT_max_ (consistent with many terrestrial arthropods, Chown and Nicolson, 2004; Fig. 3). Likewise, evidence of low vulnerability being reinforced across the two traits, i.e. individuals that exhibited both high CT_max_ and low RMR *Q*_10_ values, was only detected at the 3rd larval instar and no other developmental stage (Table 3).

While there is already a growing appreciation among vulnerability assessment approaches to consider multiple physiological traits (e.g. Shah *et al.,* 2023), their variance and covariance across ontogeny are not well understood for many taxa, especially among terrestrial organisms (Klockmann and Fischer, 2017). Indeed, it remains to be seen whether the rugged landscape of ontogenetic changes in vulnerability that we found for the painted lady butterfly is rare or common among terrestrial ectotherms. Further, we do not yet have a complete picture of ontogenetic changes in vulnerability even for the painted lady butterfly, as our current methods were unable to assess vulnerability at the earliest developmental stages. We do not know if ontogenetic changes in vulnerability from the egg stage through the 2nd larval instar are also quite rugged as they were for the 3rd through 5th larval instars, or if they are consistent with patterns from other studies showing the most severe thermal bottlenecks at the earliest developmental stages (e.g. Levy *et al.,* 2015). Even so, among the stages we were able to measure, the shift in vulnerability from the most to least vulnerable stages was of an ecologically relevant magnitude (e.g. compared with other intra- and inter-specific sources of variation in climate vulnerability traits, Diamond and Yilmaz, 2018). CT_max_ shifted by a magnitude of over 2°C, and RMR *Q*_10_ shifted by a factor of over 0.8 (Fig. 2).

Interestingly, we found that the rugged vulnerability landscape was fairly robust to changes in developmental acclimation temperature. Specifically, we found no effect of acclimation temperature on RMR *Q*_10_, consistent with the mixed support and lack of support for this relationship from other studies (Havird *et al.,* 2020). For CT_max_, although we found evidence of beneficial thermal acclimation consistent with findings from other ectothermic systems (Angilletta, 2009), acclimation temperature had little effect on the patterns of vulnerability across ontogeny. The one exception was that the 30°C treatment appeared to ameliorate the large drop in CT_max_ in the 5th instar compared with animals reared under the 20°C treatment (Fig. 1C,D). Thus, while our study suggests that vulnerability assessments might be complicated by erratic changes in vulnerability across ontogeny, developmental acclimation might not have appreciable interactive effects with these patterns. This could simplify the range of environmental conditions under which ontogenetic variation in vulnerability needs to be assessed.

### Biological rather than methodological factors likely drive erratic ontogenetic changes in vulnerability

First, it is highly unlikely that the low vulnerability of 3rd instar larvae is driven by experimental artefacts of the time lag between air temperature and core body temperature. The assessment of RMR *Q*_10_ values had a 15-minute acclimation period within each acute test temperature prior to the start of respirometry recordings. Although the air-to-body temperature lag is undoubtedly present for the assessment of CT_max_ with a 1°C min^−1^ rate of temperature increase (Oyen and Dillon, 2018), the expectation would be that the small body mass of 3rd instar larvae would cause them to heat more quickly and thus have lower CT_max_. However, 3rd instar larvae have the highest CT_max_ of any stage tested (Fig. 1C,D).

While RMR *Q*_10_ is low during the 3rd larval instar, essentially by default owing to small body mass at that stage (Fig. 1E,F), the fact that CT_max_ reaches its highest stage-specific value during the 3rd larval instar (and despite the small body mass of this stage; Fig. 1C,D) could reflect adaptive decoupling across developmental stages (Moore and Martin, 2019). Quantitative genetic, genomic association, and RNAi knockout studies (Freda *et al.,* 2017, 2022) provide strong evidence for decoupling of thermal physiological traits across ontogeny, so the highly variable nature of CT_max_ across ontogeny in the painted lady butterfly is unsurprising. Yet the question remains regarding why 3rd instar larvae would need to be so heat tolerant. Here, it is worth considering the factors that ameliorate or elevate exposure to stressfully high temperatures across the life cycle in the painted lady butterfly. Eggs and early instar larvae can be protected by leaf boundary layer effects (Kingsolver *et al.,* 2011), though we do not have heat tolerance data for these stages to assess potentially relaxed selection. Third instar larvae are sufficiently large to no longer be protected by those boundary effects (Woods, 2013), but are still small enough to potentially be limited in locomotor capacity and escape speed when seeking thermal refuge (Brackenbury, 1999). By contrast, 4th and 5th instar larvae are larger with potentially greater locomotor capacity, as are adults with the additional capability of flight and shifting their wing positioning to thermoregulate (Kingsolver, 1985).

### Relevance for conservation of seasonal migrants

There are some data to suggest that seasonally migrating species exhibit reduced tolerances of thermal extremes compared with resident species, potentially owing to the avoidance of extreme temperatures through movement. Kimura and Beppu (1993), found that adults of *Drosophila curviceps*, a species that engages in seasonal migration upslope to avoid summer high temperatures in lowland sites, exhibited worse heat tolerance compared with non-migrating congeners (*D. albomicans* and *D. immigrans*) in lowland sites. Under climate change, while some migratory species are capable of shifting the timing and locations of their migration stops to keep within their historical niches (Sparks *et al.,* 2005), migratory species that lack such flexibility could be more vulnerable than resident species owing to their reduced climatic tolerances. However, this expectation ignores ontogenetic variation in climatic vulnerability, and thus it remains an open question whether migratory species are more or less physiologically vulnerable to climate change compared with resident species.

Although we do not know whether rugged ontogenetic landscapes of vulnerability are typical of migratory species, data from one other migratory species support this hypothesis. The long-distance seasonally migrating monarch butterfly exhibited a similarly rugged landscape of vulnerability across ontogeny as to what we found with the painted lady butterfly. York and Oberhauser (2002) assessed mortality of chronic thermal stress (36°C) applied at each developmental stage from the 1st through the 5th larval instar and at the pupal stage. They found that while 1st, 3rd and 5th instar larvae and pupae that experienced chronic thermal stress had significantly higher mortality compared with larvae and pupae reared under control conditions (27°C), there were no significant differences between stressed and control organisms at the 2nd or 4th larval instars. Although these results describe ontogenetic variation in survival following thermal challenges rather than physiological indicator traits *per se* such as thermal tolerance or thermal sensitivity, they nonetheless support our findings of both bumpy and abrupt changes in vulnerability across the life cycle. Furthermore, for the imperilled monarch butterfly, this rugged landscape of ontogenetic vulnerability has important implications for understanding responses to climate change. Climate is a critical driver of population size in this species: models of monarch population size fluctuations based on long-term monitoring data revealed that breeding-season weather had substantially higher relative importance (the amount of explained variance attributable to particular factors) compared with milkweed host plant availability and migration between breeding and overwintering grounds (Zylstra *et al.,* 2021). Linking these climate-population associations with ontogenetic changes in physiological traits could help to refine conservation plans for this species by identifying viability bottlenecks in the migration process that arise from the co-occurrence of unfavourable climatic conditions and stage-dependent climate vulnerability.

Another migratory species, the budworm, *Helicoverpa punctigera*, has also been studied for ontogenetic variation in heat tolerance and thermal sensitivity of metabolic rate (Bawa *et al.,* 2021). However, because only 5th (final) instar larvae, pupae, and adults were measured, it is difficult to assess the ruggedness of changes over the life cycle (see also Régnier *et al.,* 2023). In contrast to the migratory painted lady and monarch butterflies, the year-round resident squinting bush brown butterfly, *Bicyclus anynana*, exhibited increases in vulnerability (based on LT_50_, the temperature at which 50% of individuals died following exposure to acute heat stress) among progressively earlier pre-metamorphic stages (Klockmann *et al.,* 2017). Across the entire life cycle (eggs, 1st through 5th instar larvae, pupae and adults), vulnerability closely tracked changes in body mass for this species.

It is unclear why ontogenetic vulnerability to climate might be especially rugged for migratory insect species. Given the relatively large number and diversity of long-distance and/or seasonally migrating insects including some species of butterflies, moths, dragonflies, and locusts (Chapman *et al.,* 2015; Satterfield *et al.,* 2020), there is an opportunity to use comparative approaches to explore the patterns and mechanisms underlying ontogenetic changes in physiological traits in this group. Yet regardless of whether rugged ontogenetic landscapes of vulnerability are more common for migratory species, the fact of their existence suggests that forecasting that ignores stage-specific trait variation might provide inaccurate estimates of vulnerability.

### Study limitations and future directions

One limitation of our study involves the use of domesticated populations of painted lady butterflies. Inadvertent selection and evolution of faster development and larger body mass have been documented in other domesticated Lepidoptera species, including the tobacco hornworm, *Manduca sexta* (Kingsolver *et al.,* 2009). Although this process can shift the overall distribution of body mass, this would not impact our focal comparisons of differences between developmental stages and the role of body mass in shaping ontogenetic shifts in physiology. Similarly, there might be direct effects of evolution in a constant thermal environment on physiological traits. However, these effects might be minimal, as work in zebrafish showed no evidence of loss of CT_max_ between wild and domesticated populations, nor of the duration of domestication on CT_max_ trait values (Morgan *et al.,* 2019). Even in the case of domestication-caused evolution of physiological traits, the effect would likely be to dampen the differences between developmental stages, as all stages would experience the same thermal conditions. Our results would then be conservative underestimates of variation in among-stage responses. A second limitation of our study concerns the need to test the earliest developmental stages including the egg stage and the 1st and 2nd larval instars to be able to evaluate the hypothesis that thermal bottlenecks occur in the earliest developmental stages. These data could feasibly be collected using alternative techniques to ours such as static temperature treatments and fitting of thermal performance curves combined with more sensitive respirometry techniques (e.g. stop-flow) for small-bodied organisms (Lighton and Halsey, 2011; Jørgensen *et al.,* 2021).

Another caveat, though one not particular to our study, concerns the ecological relevance of thermal vulnerability indices. Although indices such as CT_max_ and RMR *Q*_10_ provide very general estimates of relative differences in vulnerability, there are a number of ecological factors that can further shape thermal vulnerability indices themselves and environmental exposure of the organism to thermal stress (Clusella-Trullas *et al.,* 2021). For example, in *Drosophila melanogaster*, while the adult stage has been shown to have the greatest basal heat resistance, the more sessile stages of larvae and pupae have relatively greater heat hardening capacity (Moghadam *et al.,* 2019). Understanding the interaction between physiology and climate exposure across ontogeny in the wild (Pincebourde and Casas, 2015), and additionally, the potential for effects to carry over across developmental stages (Del Rio *et al.,* 2021; Pottier *et al.,* 2022b), therefore remain key future priorities.

## Conclusions

Our study provides evidence of erratic changes in climate vulnerability across the life cycle of a long-distance seasonally migrating butterfly. Although we found that the acute thermal sensitivity of metabolic rate tracked ontogenetic changes in body mass, heat tolerance was decoupled from ontogenetic changes in body mass, indicating that body mass cannot safely be used as a proxy of ontogenetic variation in vulnerability for all physiological traits. Further, the very abrupt changes in vulnerability we observed between progressive developmental stages indicates that autocorrelation of sequentially adjacent stages cannot be assumed. In consequence, vulnerability assessments that rely on physiological traits but which fail to consider changes across the entire life cycle might run the risk of severely misestimating vulnerability.

## Supplementary Material

Web_Material_coad058
