# Supplementary material for: Life cycle complexity and body mass drive erratic changes in climate vulnerability across ontogeny in a seasonally migrating butterfly
Source: Conserv Physiol. 2023 Aug 12;11(1):coad058. doi: 10.1093/conphys/coad058 (PMC10401068; doi:10.1093/conphys/coad058)
Supplement: Web_Material_coad058 [file web_material_coad058.pdf]

Supporting Information

Table S1. Total number of individuals and number of families (in parentheses) reared under the 20 °C or 30 °C developmental acclimation temperature treatments and assessed for physiological traits (both CT_{max} and RMR Q_{10} unless otherwise indicated) in phase 1 and 2 of the experiment. Note that due to a respirometry equipment malfunction, three recordings for animals in the 30 °C acclimation treatment were not usable, and thus RMR Q_{10} values could not be calculated.

**Experiment phase**	**Developmental stage**	**20 °C developmental acclimation**<br><br>**CT_{max}, total number of individuals (Number of families)**	**20 °C developmental acclimation**<br><br>**RMR Q_{10}, total number of individuals (Number of families)**	**30 °C developmental acclimation**<br><br>**CT_{max}, total number of individuals (Number of families)**	**30 °C developmental acclimation**<br><br>**RMR Q_{10}, total number of individuals (Number of families)**
1	3rd instar	33 (11)	33 (11)	38 (11)	38 (11)
	5th instar	33 (11)	33 (11)	31 (10)	31 (10)
	Pupa*	–	34 (11)	–	30 (11)
	Adult**	31 (11)	–	39 (7)	–

2	3rd instar	15 (5)	15 (5)	15 (5)	15 (5)
	4th instar	31 (5)	31 (5)	32 (5)	32 (5)
	Adult	30 (5)	30 (5)	31 (5)	28 (5)

* RMR Q_{10} measurements only

** CT_{max} measurements only

Table S2. Statistical significance (test statistics and *P*-values) of predictors from linear mixed effects models of body mass and age as functions of developmental stage, including developmental acclimation temperature, stage, and their interaction. For acclimation temperature ndf = 1; for stage and its interaction with acclimation temperature, ndf = 4. The ddf = 867 for the model of body mass and the ddf = 1377 for the model of age. Significant effects ($P < 0.05$) have bolded *P*-values.

**Response**	**Term**	***F***	***P***
Body mass	Acclimation	6.43	**0.0114**
	Stage	261	**<0.0001**
	Acclimation × Stage	22.5	**<0.0001**
Age	Acclimation	6150	**<0.0001**
	Stage	4560	**<0.0001**
	Acclimation × Stage	2150	**<0.0001**

Table S3. Estimated marginal means and standard errors for body mass (natural log transformed, mg) and age at a given developmental stage (days) across the two developmental acclimation temperature treatments from the models presented in Table S2.

**Response**	**Acclimation temperature**	**Stage**	**Estimate**	**SE**
Body mass	20 °C	3rd instar	1.83	0.0304
		4th instar	3.46	0.054
		5th instar	4.95	0.0499
		Pupa	6.44	0.0476
		Adult	5.78	0.0598
	30 °C	3rd instar	1.91	0.0307
		4th instar	4.1	0.0409
		5th instar	5.19	0.0486
		Pupa	6.39	0.0491
		Adult	5.72	0.0624

Age	20 °C	3rd instar	14.1	0.129
		4th instar	17.9	0.137
		5th instar	22.2	0.143
		Pupa	30.9	0.155
		Adult	43.2	0.157
	30 °C	3rd instar	5.52	0.13
		4th instar	7.38	0.137
		5th instar	9.15	0.143
		Pupa	12.9	0.152
		Adult	17.9	0.154

Table S4. Pairwise post-hoc comparisons for the differences in physiological traits, CT_{max} and RMR Q_{10}, between developmental stages from mass-dependent models for a given developmental acclimation temperature. Contrasts are given such that the later developmental stage is subtracted from the earlier stage. For stage contrast abbreviations, 3, 4, and 5 correspond with their respective larval instars, P indicates pupae, and A indicates adults. For CT_{max}, ddf = 336; for RMR Q_{10}, ddf = 325. Significant contrasts ($P < 0.05$) have bolded *P*-values.

**Response**	**Acclimation temperature**	**Contrast**	**Estimate**	**SE**	***t***	***P***
CT_{max}	20 °C	3 - 4	1.49	0.244	6.1	**<0.0001**
		3 - 5	2.13	0.236	9.02	**<0.0001**
		3 - A	1.22	0.201	6.06	**<0.0001**
		4 - 5	0.641	0.269	2.39	0.0818
		4 - A	-0.269	0.232	-1.16	0.652
		5 - A	-0.91	0.227	-4.01	**0.000439**
	30 °C	3 - 4	2.15	0.238	9.03	**<0.0001**
		3 - 5	1.48	0.236	6.27	**<0.0001**

		3 - A	1.26	0.191	6.62	**<0.0001**
		4 - 5	-0.667	0.271	-2.46	0.0677
		4 - A	-0.883	0.226	-3.91	**0.000642**
		5 - A	-0.216	0.227	-0.951	0.777
RMR Q_{10}	20 °C	3 - 4	-0.313	0.106	-2.96	**0.0268**
		3 - 5	-0.664	0.101	-6.58	**<0.0001**
		3 - P	-0.352	0.1	-3.52	**0.00444**
		3 - A	-0.684	0.107	-6.42	**<0.0001**
		4 - 5	-0.351	0.118	-2.98	**0.0253**
		4 - P	-0.0394	0.117	-0.337	0.997
		4 - A	-0.371	0.113	-3.27	**0.0104**
		5 - P	0.312	0.108	2.88	**0.0344**
		5 - A	-0.0202	0.118	-0.171	1

		P - A	-0.332	0.118	-2.82	**0.0407**
	30 °C	3 - 4	-0.783	0.103	-7.59	**<0.0001**
		3 - 5	-0.81	0.101	-8.03	**<0.0001**
		3 - P	-0.434	0.102	-4.25	**0.000273**
		3 - A	-0.732	0.107	-6.81	**<0.0001**
		4 - 5	-0.0279	0.119	-0.236	0.999
		4 - P	0.349	0.119	2.92	**0.0306**
		4 - A	0.0508	0.115	0.442	0.992
		5 - P	0.377	0.114	3.31	**0.00899**
		5 - A	0.0787	0.122	0.644	0.968
		P - A	-0.298	0.123	-2.42	0.113

Table S5. Pairwise post-hoc comparisons for the differences in physiological traits, CT_{max} and RMR Q_{10}, between developmental stages mass-independent models for a given developmental acclimation temperature. Contrasts are given such that the later developmental stage is subtracted from the earlier stage. For stage contrast abbreviations, 3, 4, and 5 correspond with their respective larval instars, P indicates pupae, and A indicates adults. For CT_{max}, ddf = 335; for RMR Q_{10}, ddf = 324. Significant contrasts ($P < 0.05$) have bolded *P*-values.

**Response**	**Acclimation temperature**	**Contrast**	**Estimate**	**SE**	***t***	***P***
CT_{max}	20 °C	3 - 4	0.968	0.393	2.46	0.0678
		3 - 5	1.14	0.633	1.8	0.274
		3 - A	0.0291	0.735	0.0395	1
		4 - 5	0.173	0.387	0.447	0.97
		4 - A	-0.939	0.461	-2.04	0.177
		5 - A	-1.11	0.256	-4.34	**0.000113**
	30 °C	3 - 4	1.39	0.51	2.72	**0.0344**
		3 - 5	0.46	0.651	0.706	0.895

		3 - A	0.121	0.706	0.172	0.998
		4 - 5	-0.927	0.312	-2.97	**0.0166**
		4 - A	-1.27	0.321	-3.95	**0.000557**
		5 - A	-0.338	0.238	-1.42	0.486
RMR Q_{10}	20 °C	3 - 4	-0.0365	0.169	-0.216	1
		3 - 5	-0.112	0.274	-0.41	0.994
		3 - P	0.451	0.385	1.17	0.768
		3 - A	-0.036	0.323	-0.111	1
		4 - 5	-0.0757	0.168	-0.45	0.992
		4 - P	0.488	0.266	1.84	0.355
		4 - A	0.000575	0.207	0.00278	1
		5 - P	0.563	0.159	3.54	**0.0042**
		5 - A	0.0763	0.127	0.6	0.975

		P - A	-0.487	0.134	-3.64	**0.00288**
	30 °C	3 - 4	-0.358	0.226	-1.58	0.51
		3 - 5	-0.227	0.288	-0.789	0.934
		3 - P	0.371	0.386	0.961	0.872
		3 - A	-0.0788	0.326	-0.242	0.999
		4 - 5	0.13	0.135	0.965	0.871
		4 - P	0.729	0.207	3.51	**0.0046**
		4 - A	0.279	0.156	1.78	0.385
		5 - P	0.598	0.153	3.91	**0.00106**
		5 - A	0.148	0.127	1.17	0.768
		P - A	-0.45	0.138	-3.27	**0.0103**

Table S6. Statistical tests of potential interaction effects between sex, body mass, and developmental acclimation temperature treatment on adult CT_{max} and RMR Q_{10}, and pupal RMR Q_{10}. The ndf for all terms was 1, and the ddf for adult CT_{max} and RMR Q_{10}, and pupal RMR Q_{10} were 86, 35, and 39, respectively. Significant effects ($P < 0.05$) have bolded *P*-values.

**Response**	**Stage**	**Term**	***F***	***P***
CT_{max}	Adult	Body mass	1.33	0.252
		Acclimation	4.78	**0.0315**
		Sex	0.0101	0.92
		Acclimation × Body mass	2.11	0.15
		Body mass × Sex	0.038	0.846
		Acclimation × Sex	0.0646	0.8
		Acclimation × Body mass × Sex	0.0208	0.886
RMR Q_{10}	Pupa	Body mass	0.259	0.613

		Acclimation	1.44	0.237
		Sex	1.44	0.237
		Acclimation × Body mass	1.46	0.234
		Body mass × Sex	0.767	0.386
		Acclimation × Sex	2.22	0.145
		Acclimation × Body mass × Sex	1.52	0.225
	Adult	Body mass	0.1	0.754
		Acclimation	0.088	0.768
		Sex	0.0656	0.799
		Acclimation × Body mass	0.0121	0.913
		Body mass × Sex	0.0771	0.783
		Acclimation × Sex	0.13	0.72

		Acclimation × Body mass × Sex	0.0284	0.867
